# Examination of Neurofilament Light Chain Serum Concentrations, Physical Activity, and Cognitive Decline in Older Adults

**DOI:** 10.1001/jamanetworkopen.2022.3596

**Published:** 2022-03-22

**Authors:** Pankaja Desai, Klodian Dhana, Charles DeCarli, Robert S. Wilson, Elizabeth A. McAninch, Denis A. Evans, Kumar B. Rajan

**Affiliations:** 1 Rush Institute for Healthy Aging, Rush University Medical Center, Chicago, Illinois; 2 Department of Neurology, University of California at Davis; 3 Rush Alzheimer’s Disease Center, Rush University Medical Center, Chicago, Illinois

## Abstract

**Importance:**

Little is known about the association of serum neurofilament light chain (NfL) concentrations and physical activity with the rate of cognitive decline in older adults.

**Objective:**

To examine the association of physical activity and NfL concentrations with cognitive decline in older adults over time.

**Design, Setting, and Participants:**

This cohort study used data from the Chicago Health and Aging Project (CHAP), a population-based cohort study that recruited participants through door-to-door census in 4 Chicago-area communities and collected data between 1993 and 2012 in cycles of 3 years. Participants in CHAP who had 2 or more cognitive function assessments and at least 1 blood sample collected for NfL measurement were selected for inclusion in the current study. Data were analyzed from January to December 2021.

**Exposures:**

Self-reported physical activity (minutes per week) and serum NfL concentration (pg/mL).

**Main Outcomes and Measures:**

Associations of baseline physical activity and NfL concentrations with changes in global cognitive function over time as evaluated using the East Boston Memory Test for episodic memory, the Symbol Digit Modalities Test for perceptual speed, and the Mini-Mental State Examination. Mixed-effects regression analyses were conducted to examine associations at baseline and longitudinally.

**Results:**

The study sample included 1158 participants (695 [60%] African American; 728 [63%] female), with a mean (SD) age of 77.4 (6.0) years and a mean educational level of 12.6 (3.5) years. Among participants with high NfL concentrations (>25 pg/mL), those who engaged in medium physical activity (<150 minutes per week) had a 12% slower rate of global cognitive decline (SD units, or β, –0.065; 95% CI, −0.099 to −0.032) and participants who engaged in high physical activity (≥150 minutes per week) had a 36% slower rate of decline (β, –0.048; 95% CI, −0.080 to −0.016) than did participants with low physical activity (no reported participation) (β, –0.075; 95% CI, −0.108 to −0.041). For participants with low NfL concentrations (≤25 pg/mL), those who took part in medium physical activity had 43% slower global cognitive decline (β, –0.025; 95% CI, −0.043 to −0.007) and individuals who participated in high physical activity had 30% slower decline (β, –0.031; 95% CI, −0.048 to −0.014) than did those who participated in low physical activity (β, –0.046; 95% CI, −0.066 to −0.025).

**Conclusions and Relevance:**

The findings suggest that physical activity is associated with diminished cognitive decline among older adults with increased serum NfL concentrations. The results support the potential use of blood biomarkers in measuring the benefits of health behaviors, such as physical activity, and early intervention for older adults at risk for cognitive decline.

## Key Points

**Question:**

Is physical activity associated with lower rates of cognitive decline in older adults with higher serum concentrations of neurofilament light chain (NfL)?

**Findings:**

In this cohort study of 1158 older adults, compared with low physical activity, medium physical activity was associated with a 43% slower rate of cognitive decline and high physical activity with a 30% slower rate in participants with low NfL concentrations. Compared with low physical activity, medium and high physical activity were associated with a 12% and 36% slower rate of cognitive decline, respectively, in participants with high NfL concentrations.

**Meaning:**

The findings suggest that engagement in medium and high physical activity is associated with slower cognitive decline compared with low physical activity among older adults with varying levels of serum NfL concentrations.

## Teaser

This cohort study uses data from the Chicago Health and Aging Project to examine associations between physical activity and cognitive decline by serum concentrations of neurofilament light (NfL) chain among older adults.

## Introduction

Determining axonal damage is critical to ensure accurate diagnosis of neurodegenerative conditions. Neurofilament light chain (NfL) is a protein located in the cytoplasm of neurons and expressed when axons are myelinated.^1^ Levels of NfL in the cerebrospinal fluid are generally well correlated with blood NfL levels.^2^ Levels of NfL in the blood increase in proportion to the amount of damage to axons.^1^ New developments in immunoassay methods provide an opportunity for detection of low levels of NfL in blood, which is beneficial for diagnosis, monitoring, and determining symptom severity.^1,3,4,5^ Increased levels of NfL are usually but not consistently associated with poor cognition or neurological diseases.^4^ Individuals with dementia tend to have increased NfL levels compared with persons who have mild cognitive impairment.^4,6^ High NfL levels are associated with increased likelihood of poor cognitive test performance.^4^ In addition, NfL levels may represent the integrity of white matter in the brain.^5^ Serum NfL levels may be associated with declines in cognition, Alzheimer disease (AD) that is sporadic, and structural transformations in the brain.^3^

Our primary research question was the following: does NfL moderate the association between physical activity and cognitive decline? Limited information exists about the associations between blood biomarkers, physical activity, and cognitive function. Prior work showed that physical activity was associated with slower cognitive decline in persons with both high and low concentrations of total tau, a different blood biomarker for AD.^7^ Physical activity is a modifiable factor associated with risk for AD, is inversely associated with cognitive decline, and may help prevent cognitive decline and impede the course of AD.^8,9^ The long preclinical phase that is characteristic of AD provides the opportunity for health behavior intervention, including increasing physical activity engagement and maintenance.^3,10,11^ In addition, measuring NfL levels in blood offers an efficient way to evaluate cognitive decline and an opportunity for initial detection of AD pathology.^3,12^ NfL has been shown to be a blood biomarker for neurodegeneration.^13^ This study evaluated whether physical activity moderated the association between NfL levels and cognitive function over time in participants in the Chicago Health and Aging Project (CHAP).^14^ Although much is unknown about blood biomarkers for AD, we assumed that individuals with high concentrations of NfL would be more likely to experience more rapid cognitive decline than would those with low NfL concentrations. The purpose of this study was to examine whether among individuals with high NfL concentrations, a factor that may be associated with cognitive decline, engagement in more physical activity is associated with a slowed rate of cognitive decline over time and thus with reduced disease severity and maintained quality of life.

## Methods

This cohort study used data from CHAP, which recruited participants older than 65 years who were African American or White by door-to-door census in 4 Chicago-area communities (the population samples of Hispanic participants [1.3%] and participants of other races and ethnicities [0.3%] were small, which did not provide enough statistical power to examine racial and ethnic differences). Data collection among CHAP participants involved in-home interviews and was conducted in 3-year cycles from October 8, 1993, to October 18, 2012. Data analysis for the current study was conducted from January to December 2021. The CHAP study and the current study were approved by the Rush University Medical Center Institutional Review Board. All study participants provided written informed consent. This study followed the Strengthening the Reporting of Observational Studies in Epidemiology (STROBE) reporting guideline.

Clinical evaluations, including blood sample collection, were completed with a stratified random sample of approximately one-third of CHAP participants. Clinical evaluations occurred at baseline and during each follow-up measurement, and cognitive tests were administered at baseline and during each follow-up measurement. A total of 11 600 blood samples were collected from 5696 participants from 1993 to 2012. Immunoassays were conducted on only 3000 samples because of budgetary limitations.^3^ Race was determined using the 1990 US census questions.^15^ Participants were asked to report the following conditions: stroke, cancer, high blood pressure, heart problems (a heart attack or coronary, or coronary thrombosis, or coronary occlusion, or myocardial infarction), diabetes, or broken or fractured hip. A variable for these conditions was created by summing the number of conditions among participants. Study participants did not have a diagnosis of AD and were aged 65 years or older at baseline.^14^

### Measurement of Serum NfL Concentration

Study team members used dry ice to move blood samples to the Rush University Medical Center Biorepository freezer (−80 °C). Unthawed samples were sent to Quanterix Corporation in Billerica, Massachusetts, in 2019, and Quanterix’s single, bead-based HD-X molecular immunoassay platform and the Neurology 4Plex A kit were used to assay NfL in duplicates. For this analysis, mean NfL concentrations of duplicate measurements were used, with a coefficient of variation of 3.0%. The NfL concentrations were categorized into 2 groups based on prior work: low (≤25.5 pg/mL) and high (>25.5 pg/mL).^3,16^

### Physical Activity

Self-reported physical activity was measured using the 1985 US Health Interview Survey items.^17,18^ Participants were asked to indicate the frequency (instances) and duration (minutes) of participation in walking for exercise, jogging or running, gardening or yard work, dancing, calisthenics or general exercise, golf, bowling, bicycle riding, swimming or water exercises, other exercises, sports, or physically active hobbies in the past 14 days.^17,18^ The total time of physical activity per week, in minutes, was determined by multiplying the number of instances of physical activity by the duration and dividing by 2 (ie, 7 days per week) for each item and summing across all items. Physical activity was divided into 3 groups: little activity (participant responded to at least 4 items on the physical activity measure, and all responses indicated no participation [0 minutes per week]), medium activity (<150 minutes of activity participation per week), and high activity (≥150 minutes of activity participation per week). The cut point of 150 minutes per week was used based on *Physical Activity Guidelines for Americans* (2nd edition).^19^

### Cognitive Function Battery

Global cognitive function was evaluated during in-home interviews through the East Boston Memory Test: Immediate Recall and Delayed Recall (episodic memory), the Mini-Mental State Examination (MMSE), and the Symbol Digit Modalities Test (modified, oral version) (perceptual speed).^20,21,22,23^ Baseline mean scores and SDs of the total CHAP sample were used to calculate *z* scores for each test. Mean *z* scores of all tests were obtained to calculate global cognitive function.^24^

### Statistical Analysis

SAS, version 9.4 (SAS Institute Inc) was used to conduct statistical analysis. Descriptive statistics were calculated for the sample at baseline. Mixed-effects regression analyses were conducted starting with the date of the first blood sample collection and at all following interviews to examine the associations at baseline and longitudinally between independent variables (baseline physical activity and NfL level) and dependent variables (global cognitive function and individual tests [episodic memory, perceptual speed, and MMSE score]). Models included all available measurements of physical activity and NfL and adjusted for age, race, sex, educational level, chronic medical conditions, and presence of the apolipoprotein E (*APOE*) ε4 allele and the interactions of each with time. Models were composed of person-specific intercepts and slopes and had an unstructured correlation matrix structure. R, version 4.0.3 (R Project for Statistical Computing) was used for developing plots. Hypothesis tests were 2-sided. The a priori level of significance was *P* < .05.

## Results

### Descriptive Analysis

The baseline study sample included 1158 participants who provided at least 1 blood sample and completed at least 2 global cognitive function outcome assessments (mean [SD] age, 77.4 [6.0] years; 695 [60%] African American; 728 [63%] female; mean [SD] educational level, 12.6 [3.5] years) (Table 1). The mean (SD) amount of physical activity per week was 170.78 (269.48) minutes. The geometric mean of NfL concentrations was 26.1 pg/mL (95% CI, 25.2-27.1 pg/mL). The lowest observed value of detection for NfL concentrations was 0.99 pg/mL.

**Table 1.** Baseline Characteristics of the Study Participants

Characteristic	Participants (N = 1158)^a^
Age, mean (SD), y	77.4 (6.0)
Educational level, mean (SD), y	12.6 (3.5)
Sex	
Female	728 (63)
Male	430 (37)
Race	
African American	695 (60)
White	463 (40)
*APOE* ε4 allele	393 (34)
Composite physical activity, mean (SD), min/wk	170.78 (269.48)
Physical activity category	
Low^b^	356 (31)
Medium^c^	400 (35)
High^d^	402 (35)
Serum NfL concentration, geometric mean (95% CI), pg/mL	26.1 (25.2-27.1)
Serum NfL concentration category	
Low: ≤25.5 pg/mL	618 (53)
High: >25.5 pg/mL	540 (47)
Global cognitive function score, mean (SD)	0.21 (0.68)
Standardized memory score, mean (SD)	0.19 (0.83)
Standardized MMSE score, mean (SD)	0.26 (0.61)
Standardized speed score, mean (SD)	0.24 (0.90)

Abbreviations: *APOE*, apolipoprotein E; MMSE, Mini-Mental State Examination; NfL, neurofilament light chain.

^a^ Data are presented as the number (percentage) of participants unless otherwise indicated.

^b^ Participants responded to at least 4 items on the physical activity measure and reported no activity for all responses.

^c^ Less than 150 minutes per week.

^d^ At least 150 minutes per week.

### Mixed-Effects Regression Models

In mixed-effects regression models to test the interaction of physical activity with NfL with time, there were no statistically significant associations for any cognitive outcomes. The separate models for high and low NfL groups examining the association of physical activity with each cognitive outcome showed some statistically significant associations over time (Table 2). Among participants with high NfL concentrations, there was a statistically significant association between high physical activity and episodic memory (β, 0.039 [SE, 0.018]; *P* = .03) but high physical activity was not associated with perceptual speed (β, 0.027 [SE, 0.014]; *P* = .06). Among participants with low NfL concentrations, there were statistically significant associations between medium physical activity and global cognitive function (β, 0.020 [SE, 0.010]; *P* = .04), perceptual speed (β, 0.024 [SE, 0.011]; *P* = .03), and MMSE score (β, 0.027 [SE, 0.011]; *P* = .02). We conducted *t* tests to assess whether there were differences in NfL grouping (high vs low) by *APOE* ε4 allele possession (any ε4 allele vs none) and found no statistically significant differences. Therefore, we adjusted for *APOE* ε4 allele possession in models because of its established association with cognition and because we did not know its association with NfL. Models tested the interaction of *APOE* ε4 allele possession with time within NfL groupings and revealed statistically significant associations with all cognitive outcomes except for perceptual speed among participants with high NfL concentrations. Assumptions of models were determined to be sufficiently met through both analytical and graphical evaluation.

**Table 2.** Interactions of Physical Activity With Time by NfL Concentration^a^

Interaction	Low NfL^b^				High NfL^c^			
	Participants, No.	Observations, No.	β (SE)	*P* value	Participants, No.	Observations, No.	β (SE)	*P* value
Global cognition								
Medium physical activity × time^d^	619	1976	0.020 (0.010)	.04	541	1533	0.009 (0.014)	.51
High physical activity × time^e^			0.014 (0.010)	.15			0.027 (0.015)	.08
Episodic memory								
Medium physical activity × time^d^	616	1957	0.013 (0.013)	.32	535	1503	−0.002 (0.017)	.90
High physical activity × time^e^			0.007 (0.013)	.58			0.039 (0.018)	.03
Perceptual speed								
Medium physical activity × time^d^	611	1937	0.024 (0.011)	.03	510	1436	0.017 (0.013)	.21
High physical activity × time^e^			0.022 (0.011)	.05			0.027 (0.014)	.06
MMSE score								
Medium physical activity × time^d^	619	1975	0.027 (0.011)	.02	542	1534	0.026 (0.018)	.15
High physical activity × time^e^			0.020 (0.011)	.07			0.015 (0.019)	.45

Abbreviations: MMSE, Mini-Mental State Examination; NfL, neurofilament light chain.

^a^ All regression models were adjusted for age, race, sex, educational level, chronic conditions, and presence of the apolipoprotein E ε4 allele.

^b^ Serum NfL concentration of 25.5 pg/mL or less.

^c^ Serum NfL concentration greater than 25.5 pg/mL.

^d^ Less than 150 minutes per week.

^e^ At least 150 minutes per week.

### Baseline Global Cognitive Function

Table 3 shows the associations of physical activity and NfL with baseline global cognitive function. Among participants with low NfL concentrations (≤25.5 pg/mL), global cognitive function at baseline was 9% higher among participants with medium physical activity (SD units, or β, 0.476; 95% CI, 0.369-0.582) and 3% higher among those with high physical activity (β, 0.509; 95% CI, 0.411-0.608) than among those with low physical activity (β, 0.525; 95% CI, 0.407-0.642). Among participants with high NfL concentrations (>25.5 pg/mL), baseline global cognitive function was 75% higher for medium physical activity (β, 0.445; 95% CI, 0.274-0.617) and 44% higher for high physical activity (β, 0.366; 95% CI, 0.199-0.532) than for low physical activity (β, 0.255; 95% CI, 0.083-0.427). Results for individual cognitive function tests showed results mostly similar to those for global cognitive function, as indicated in the eTable in the Supplement.

**Table 3.** Association of Physical Activity With Baseline Level of Global Cognitive Function Stratified by Serum NfL Concentration^**a**^

Variable	β (95% CI)	Difference (95% CI)	Difference, %
Low NfL^b^			
Low physical activity^c^	0.525 (0.407 to 0.642)	1 [Reference]	1 [Reference]
Medium physical activity^d^	0.476 (0.369 to 0.582)	−0.049 (−0.152 to 0.054)	9
High physical activity^e^	0.509 (0.411 to 0.608)	−0.015 (−0.122 to 0.091)	3
High NfL^b^			
Low physical activity^c^	0.255 (0.083 to 0.427)	1 [Reference]	1 [Reference]
Medium physical activity^d^	0.445 (0.274 to 0.617)	0.191 (0.054 to 0.327)	75
High physical activity^e^	0.366 (0.199 to 0.532)	0.111 (−0.037 to 0.259)	44

Abbreviation: NfL, neurofilament light chain.

^a^ All regression models were adjusted for age, race, sex, educational level, chronic conditions, and presence of the apolipoprotein E ε4 allele.

^b^ Serum NfL concentration: low, 25.5 pg/mL or less; high, greater than 25.5 pg/mL.

^c^ Participants responded to at least 4 items on the physical activity measure and reported no activity for all responses.

^d^ Less than 150 minutes per week.

^e^ At least 150 minutes per week.

### Global Cognitive Decline

Table 4 shows global cognitive decline annually among participants with low and high NfL concentrations. Participants with low NfL concentrations showed a 43% slower decline for medium physical activity (β, –0.025; 95% CI, −0.043 to −0.007) and 30% slower decline for high physical activity (β, –0.031; 95% CI, −0.048 to −0.014) than for low physical activity (β, –0.046; 95% CI, −0.066 to −0.025). For participants with high NfL concentrations, global cognitive decline was 12% slower for medium physical activity (β, –0.065; 95% CI, −0.099 to −0.032) and 36% slower for high physical activity (β, –0.048; 95% CI, −0.080 to −0.016) than for low physical activity (β, –0.075; 95% CI, −0.108 to −0.041).

**Table 4.** Association of Physical Activity With Annual Rate of Global Cognitive Decline Stratified by Serum NfL Concentration^**a**^

Variable	β (95% CI)	Difference (95% CI)	Difference, %
Low NfL^b^			
Low physical activity^c^	−0.046 (−0.066 to −0.025)	1 [Reference]	1 [Reference]
Medium physical activity^d^	−0.025 (−0.043 to −0.007)	0.020 (0.001 to 0.039)	43
High physical activity^e^	−0.031 (−0.048 to −0.014)	0.014 (−0.005 to 0.034)	30
High NfL^b^			
Low physical activity^c^	−0.075 (−0.108 to −0.041)	1 [Reference]	1 [Reference]
Medium physical activity^d^	−0.065 (−0.099 to −0.032)	0.009 (−0.019 to 0.037)	12
High physical activity^e^	−0.048 (−0.080 to −0.016)	0.027 (−0.003 to 0.057)	36

Abbreviation: NfL, neurofilament light chain.

^a^ All regression models were adjusted for age, race, sex, educational level, chronic conditions, and presence of the apolipoprotein E ε4 allele.

^b^ Serum NfL concentration: low, 25.5 pg/mL or less; high, greater than 25.5 pg/mL.

^c^ Participants responded to at least 4 items on the physical activity measure and reported no activity for all responses.

^d^ Less than 150 minutes per week.

^e^ At least 150 minutes per week.

### Decline in Episodic Memory

The eTable in the Supplement describes the decline in episodic memory over time in the NfL and physical activity groups. For participants with low NfL concentrations, those with medium physical activity had a 65% slower decline (β, –0.007; 95% CI, −0.03 to 0.016) and those with high physical activity had a 35% slower decline in episodic memory (β, –0.013; 95% CI, −0.035 to 0.009) than did those with low physical activity (β, −0.020; 95% CI, −0.047 to 0.007). Among participants with high NfL concentrations, the medium physical activity group had a 5% slower decline (β, −0.046; 95% CI, −0.085 to −0.008) and the high physical activity group had an 89% slower decline (β, –0.005; 95% CI, −0.042 to 0.032) than did the low physical activity group (β, −0.044; 95% CI, −0.083 to −0.005).

### Decline in Perceptual Speed

The eTable in the Supplement shows the annual decline in perceptual speed in the NfL and physical activity categories. Individuals with low NfL concentrations had 33% slower decline with medium physical activity (β, −0.049; 95% CI, −0.070 to −0.029) and 30% slower decline with high physical activity (β, −0.051; 95% CI, −0.070 to −0.032) than with low physical activity (β, −0.073; 95% CI, −0.097 to −0.049). Participants with high NfL concentrations showed a 19% slower decline with medium physical activity (β, −0.074; 95% CI, −0.104 to −0.044) and 30% slower decline with high physical activity (β, −0.064; 95% CI, −0.093 to −0.035) than with low physical activity (β, −0.090; 95% CI, −0.121 to −0.060).

### Decline in MMSE

The eTable in the Supplement shows the decline by year in MMSE score among participants by NfL and physical activity classification. Individuals with low NfL concentrations had a 49% slower decline in the medium physical activity group (β, −0.028; 95% CI, −0.048 to −0.007) and a 36% slower decline in the high physical activity group (β, −0.034; 95% CI, −0.054 to −0.015) than in the low physical activity group (β, −0.055; 95% CI, −0.078 to −0.031). Among participants with high NfL concentrations, those with medium physical activity (β, −0.081; 95% CI, −0.125 to −0.038) had a 24% slower decline and those with high physical activity (β, −0.093; 95% CI, −0.135 to −0.051) had a 14% slower decline than those with low physical activity (β, −0.108; 95% CI, −0.151 to −0.064).

### Longitudinal Change by Physical Activity Level and NfL Concentration

The Figure shows the yearly change in each cognitive measure by physical activity level and NfL concentration. The plots show substantial declines in cognitive function among participants with low physical activity and high NfL. This group had the lowest scores over time compared with the other NfL and physical activity groups. Participants with low NfL concentrations tended to have slower rates of cognitive decline over time than did participants with high NfL concentrations. Among participants with high concentrations of NfL, those who engaged in medium and high physical activity had slower rates of cognitive decline than did those with low physical activity levels.

**Figure.**
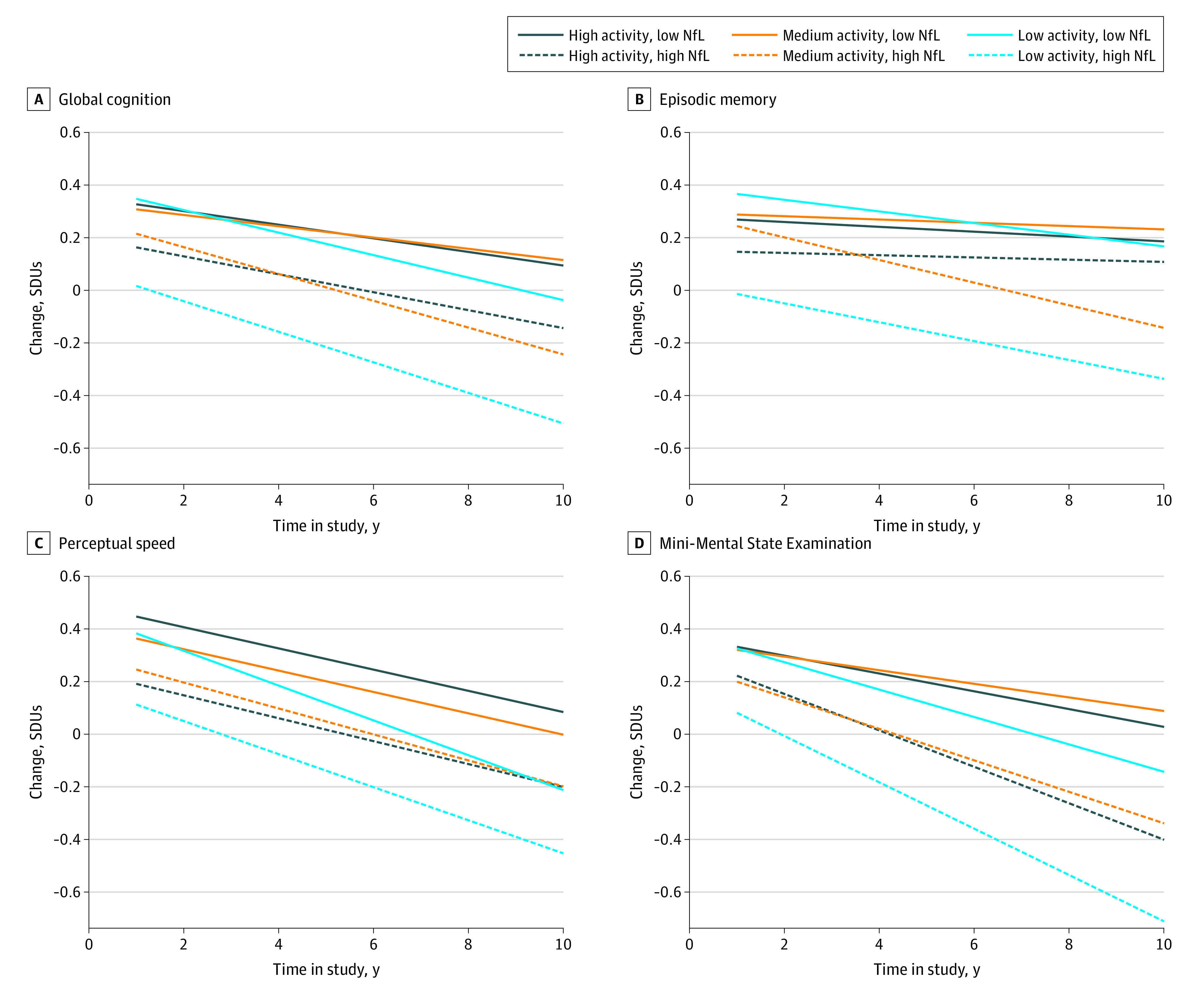
Change in Cognition Over Time by Physical Activity Level and Serum Neurofilament Light Chain (NfL) Concentration SDU indicates SD unit, or β.

## Discussion

Although engagement in physical activity is beneficial for many reasons, the results of this study suggest that the amount of physical activity associated with a reduction in the rate of cognitive decline may differ by NfL concentration. With respect to slowing the rate of cognitive decline, participating in greater levels of physical activity may be needed more for those with a high NfL concentration than for those with a low NfL concentration. This study expands on prior research conducted on serum biomarkers in CHAP.^3^ Examining the associations between AD biomarkers and health behaviors such as physical activity is a new area of research.^25^ We found only 1 study, the Multidomain Alzheimer Preventive Trial (MAPT),^26^ that evaluated associations between NfL concentrations in blood and physical activity over 4 years. In that study, high NfL concentrations were associated with reductions in the benefits of physical activity on cognitive function. Investigators have called for the need to validate results and to examine the associations between physical activity and NfL concentrations in a sample that has not received intervention.^26^ The present study leveraged the longitudinal cohort design and population-based sample of CHAP to address this need.

Previous research found that decreases in the amyloid β 42 to amyloid β 40 ratio and increases in NfL concentration, both in plasma, were associated with increased cognitive decline and decreased gait speed over time.^27^ Prior work has also shown an association between greater plasma-measured NfL concentrations and increased time in bed obtained using bedtimes and total sleep time at night through the Pittsburgh Sleep Quality Index (Dutch version), which is a self-report measure.^28,29^ This finding is consistent with the results of our study, which showed that participants with less activity performed poorly on cognitive function tests. Conversely, high-intensity interval training was associated with decreased plasma-measured NfL concentrations in individuals with multiple sclerosis.^30^ A review conducted by Ramani and colleagues^4^ revealed that the association between NfL and cognition required further confirmation in studies accounting for participant differences, neurological condition, and methods. The current study contributes to this knowledge gap by longitudinally evaluating the association between NfL concentration and cognitive function in a large sample of both African American and White older adults.

### Strengths and Limitations

This study has strengths. The study included a large, population-based sample with members of 2 racial groups. Serum biomarkers were collected, and at least 2 measurement points of cognitive function were used.

 This study also has limitations. The physical activity measure used was a self-report, which is subject to recall and social desirability biases.^31^ Intensity of physical activity was not built into the self-report measure. The range of minutes per week of total physical activity was large. A subset of the CHAP sample was used for this analysis; thus, selection bias was a limitation. Several racial and ethnic minority groups were not represented in the study. It was unclear why at times engagement in medium physical activity was associated with a slower decline rate in cognitive function than was participation in high physical activity. Limited information exists regarding the serum NfL measures and measurements that may be indicative of peripheral conditions not considered, such as vascular risk factors, osteoarthritis, or peripheral neuropathy. We also assumed that increased concentrations of NfL would be associated with a greater rate of cognitive decline or an increased likelihood of developing AD. However, these associations were not established. The models did not adjust for peripheral neuropathy, posttraumatic stress disorder, and other neurological and/or trauma conditions. The capacity to engage in physical activity is associated with nonbrain systems as well, which may not have been accounted for in this analysis. Participating in greater levels of physical activity may be helpful or harmful depending on factors such as functional status, comorbidities, disease severity, and environment.

## Conclusions

In this cohort study, physical activity was associated with a diminished rate of cognitive decline among older adults with increased serum NfL concentrations. Future research should examine other health behaviors; physical activity types, such as aerobic or strengthening activities; additional biomarkers; and outcomes, such as magnetic resonance imaging of the brain. Blood biomarkers may serve as a resourceful tool for evaluating AD risk and for tailoring interventions based on risk factors and susceptibility.^3^ The study’s results may contribute to the formation of comparative effectiveness studies or adaptive trials focused on health behavior change and maintenance in population subgroups with longitudinal measurement.

## Notes

Supplement. **eTable.** Association of PA With the Rate of Decline in Individual Tests of Cognition, Stratified by Concentration of NfL

## References

[1] Gaetani L, Blennow K, Calabresi P, Di Filippo M, Parnetti L, Zetterberg H. Neurofilament light chain as a biomarker in neurological disorders. J Neurol Neurosurg Psychiatry. 2019;90(8):870-881. doi:10.1136/jnnp-2018-320106 30967444

[2] Jin M, Cao L, Dai YP. Role of neurofilament light chain as a potential biomarker for Alzheimer’s disease: a correlative meta-analysis. *Front Aging Neurosci*. 2019;11:254. doi:10.3389/fnagi.2019.00254 PMC6753203 31572170

[3] Rajan KB, Aggarwal NT, McAninch EA, . Remote blood biomarkers of longitudinal cognitive outcomes in a population study. Ann Neurol. 2020;88(6):1065-1076. doi:10.1002/ana.25874 32799383 PMC9186023

[4] Ramani S, Berard JA, Walker LAS. The relationship between neurofilament light chain and cognition in neurological disorders: a scoping review. J Neurol Sci. 2021;420:117229. doi:10.1016/j.jns.2020.117229 33243431

[5] Schultz SA, Strain JF, Adedokun A, ; Dominantly Inherited Alzheimer Network. Serum neurofilament light chain levels are associated with white matter integrity in autosomal dominant Alzheimer’s disease. Neurobiol Dis. 2020;142:104960. doi:10.1016/j.nbd.2020.104960 32522711 PMC7363568

[6] Olsson B, Portelius E, Cullen NC, . Association of cerebrospinal fluid neurofilament light protein levels with cognition in patients with dementia, motor neuron disease, and movement disorders. JAMA Neurol. 2019;76(3):318-325. doi:10.1001/jamaneurol.2018.3746 30508027 PMC6440232

[7] Desai P, Evans D, Dhana K, . Longitudinal association of total tau concentrations and physical activity with cognitive decline in a population sample. *JAMA Netw Open*. 2021;4(8):e2120398. doi:10.1001/jamanetworkopen.2021.20398 PMC8358733 34379124

[8] Rolland Y, Abellan van Kan G, Vellas B. Physical activity and Alzheimer’s disease: from prevention to therapeutic perspectives. J Am Med Dir Assoc. 2008;9(6):390-405. doi:10.1016/j.jamda.2008.02.007 18585641

[9] Sofi F, Valecchi D, Bacci D, . Physical activity and risk of cognitive decline: a meta-analysis of prospective studies. J Intern Med. 2011;269(1):107-117. doi:10.1111/j.1365-2796.2010.02281.x 20831630

[10] Rabin JS, Klein H, Kirn DR, . Associations of physical activity and β-amyloid with longitudinal cognition and neurodegeneration in clinically normal older adults. JAMA Neurol. 2019;76(10):1203-1210. doi:10.1001/jamaneurol.2019.1879 31312836 PMC6635892

[11] Sperling RA, Aisen PS, Beckett LA, . Toward defining the preclinical stages of Alzheimer’s disease: recommendations from the National Institute on Aging-Alzheimer’s Association workgroups on diagnostic guidelines for Alzheimer’s disease. Alzheimers Dement. 2011;7(3):280-292. doi:10.1016/j.jalz.2011.03.003 21514248 PMC3220946

[12] Zetterberg H. Blood-based biomarkers for Alzheimer’s disease—an update. J Neurosci Methods. 2019;319:2-6. doi:10.1016/j.jneumeth.2018.10.025 30352211

[13] Zetterberg H, Burnham SC. Blood-based molecular biomarkers for Alzheimer’s disease. *Mol Brain*. 2019;12(1):26. doi:10.1186/s13041-019-0448-1 PMC6437931 30922367

[14] Bienias JL, Beckett LA, Bennett DA, Wilson RS, Evans DA. Design of the Chicago Health and Aging Project (CHAP). J Alzheimers Dis. 2003;5(5):349-355. doi:10.3233/JAD-2003-5501 14646025

[15] US Bureau of the Census. *1990 Census of Population: General Population Characteristics, United States*. 1990-CP-1-1. US Bureau of the Census; 1992.

[16] Quanterix. Simoa assay kits: TAU. Accessed October 20, 2021. https://www.quanterix.com/simoa-assay-kits/tau/#:~:text=The%20Simoa%E2%84%A2%20Human%20Total,assay%20recognizes%20all%20tau%20isoforms

[17] McPhillips JB, Pellettera KM, Barrett-Connor E, Wingard DL, Criqui MH. Exercise patterns in a population of older adults. Am J Prev Med. 1989;5(2):65-72. doi:10.1016/S0749-3797(18)31107-3 2730794

[18] Sturman MT, Morris MC, Mendes de Leon CF, Bienias JL, Wilson RS, Evans DA. Physical activity, cognitive activity, and cognitive decline in a biracial community population. Arch Neurol. 2005;62(11):1750-1754. doi:10.1001/archneur.62.11.1750 16286550

[19] US Department of Health and Human Services. Physical Activity Guidelines for Americans. 2nd ed. US Department of Health and Human Services; 2018.

[20] Albert MS, Jones K, Savage CR, . Predictors of cognitive change in older persons: MacArthur studies of successful aging. Psychol Aging. 1995;10(4):578-589. doi:10.1037/0882-7974.10.4.578 8749585

[21] Folstein MF, Folstein SE, McHugh PR. “Mini-mental state”: a practical method for grading the cognitive state of patients for the clinician. J Psychiatr Res. 1975;12(3):189-198. doi:10.1016/0022-3956(75)90026-6 1202204

[22] Scherr PA, Albert MS, Funkenstein HH, . Correlates of cognitive function in an elderly community population. Am J Epidemiol. 1988;128(5):1084-1101. doi:10.1093/oxfordjournals.aje.a115051 3189282

[23] Smith A. Symbol Digit Modalities Test Manual. Western Psychological Services; 1982.

[24] Wilson RS, Bennett DA, Bienias JL, Mendes de Leon CF, Morris MC, Evans DA. Cognitive activity and cognitive decline in a biracial community population. Neurology. 2003;61(6):812-816. doi:10.1212/01.WNL.0000083989.44027.05 14504326

[25] Okonkwo OC, Schultz SA, Oh JM, . Physical activity attenuates age-related biomarker alterations in preclinical AD. Neurology. 2014;83(19):1753-1760. doi:10.1212/WNL.0000000000000964 25298312 PMC4239838

[26] Raffin J, Rolland Y, Aggarwal G, ; MAPT/DSA Group. Associations between physical activity, blood-based biomarkers of neurodegeneration, and cognition in healthy older adults: the MAPT study. J Gerontol A Biol Sci Med Sci. 2021;76(8):1382-1390. doi:10.1093/gerona/glab094 33864068

[27] He L, de Souto Barreto P, Aggarwal G, ; MAPT/DSA Group. Plasma Aβ and neurofilament light chain are associated with cognitive and physical function decline in non-dementia older adults. *Alzheimers Res Ther*. 2020;12(1):128. doi:10.1186/s13195-020-00697-0 PMC7545881 33032662

[28] Lysen TS, Ikram MA, Ghanbari M, Luik AI. Sleep, 24-h activity rhythms, and plasma markers of neurodegenerative disease. Sci Rep. 2020;10(1):20691. doi:10.1038/s41598-020-77830-4 33244083 PMC7692474

[29] Buysse DJ, Reynolds CF III, Monk TH, Berman SR, Kupfer DJ. The Pittsburgh Sleep Quality Index: a new instrument for psychiatric practice and research. Psychiatry Res. 1989;28(2):193-213. doi:10.1016/0165-1781(89)90047-4 2748771

[30] Joisten N, Rademacher A, Warnke C, . Exercise diminishes plasma neurofilament light chain and reroutes the kynurenine pathway in multiple sclerosis. *Neurol Neuroimmunol Neuroinflamm*. 2021;8(3):e982. doi:10.1212/NXI.0000000000000982 PMC8054957 33782190

[31] Sallis JF, Saelens BE. Assessment of physical activity by self-report: status, limitations, and future directions. Res Q Exerc Sport. 2000;71(suppl 2):1-14. doi:10.1080/02701367.2000.11082780 25680007

