# Supplementary material for: Examination of Neurofilament Light Chain Serum Concentrations, Physical Activity, and Cognitive Decline in Older Adults
Source: JAMA Netw Open. 2022 Mar 22;5(3):e223596. doi: 10.1001/jamanetworkopen.2022.3596 (PMC8941360; doi:10.1001/jamanetworkopen.2022.3596)
Supplement: Supplement. — eTable. Association of PA With the Rate of Decline in Individual Tests of Cognition, Stratified by Concentration of NfL [file jamanetwopen-e223596-s001.pdf]

Supplementary Online Content

Desai P, Dhana K, DeCarli C, et al. Examination of neurofilament light chain serum concentrations, physical activity, and cognitive decline in older adults. *JAMA Netw Open*. 2022;5(3):e223596. doi:10.1001/jamanetworkopen.2022.3596

eTable. Association of PA With the Rate of Decline in Individual Tests of Cognition, Stratified by Concentration of NfL

This supplementary material has been provided by the authors to give readers additional information about their work.

© 2022 Desai P et al. *JAMA Network Open*.

eTable. Association of PA with the Rate of Decline in Individual Tests of Cognition, Stratified by Concentration of NfL

**Episodic Memory**					
	**Estimate (95% CI)**		**Difference (95% CI)**		**% Difference**
**Low NfL**					
Low PA	-0.02	(-0.047, 0.007)	Ref		Ref
Medium PA	-0.007	(-0.03, 0.016)	0.013	(-0.012, 0.038)	65%
High PA	-0.013	(-0.035, 0.009)	0.007	(-0.018, 0.033)	35%
**High NfL**					
Low PA	-0.044	(-0.083, -0.005)	Ref		Ref
Medium PA	-0.046	(-0.085, -0.008)	-0.002	(-0.036, 0.031)	5%
High PA	-0.005	(-0.042, 0.032)	0.039	(0.004, 0.074)	89%
**Perceptual Speed**					
	**Estimate (95% CI)**		**Difference (95% CI)**		**% Difference**
**Low NfL**					
Low PA	-0.073	(-0.097, -0.049)	Ref		Ref
Medium PA	-0.049	(-0.07, -0.029)	0.024	(0.002, 0.045)	33%
High PA	-0.051	(-0.07, -0.032)	0.022	(0, 0.044)	30%
**High NfL**					
Low PA	-0.091	(-0.121, -0.06)	Ref		Ref
Medium PA	-0.074	(-0.104, -0.044)	0.017	(-0.009, 0.043)	19%
High PA	-0.064	(-0.093, -0.035)	0.027	(-0.001, 0.054)	30%
**MMSE**					
	**Estimate (95% CI)**		**Difference (95% CI)**		**% Difference**
**Low NfL**					
Low PA	-0.055	(-0.078, -0.031)	Ref		Ref
Medium PA	-0.028	(-0.048, -0.007)	0.027	(0.005, 0.048)	49%
High PA	-0.034	(-0.054, -0.015)	0.02	(-0.002, 0.042)	36%
**High NfL**					
Low PA	-0.108	(-0.151, -0.064)	Ref		Ref
Medium PA	-0.081	(-0.125, -0.038)	0.026	(-0.009, 0.062)	24%
High PA	-0.093	(-0.135, -0.051)	0.015	(-0.023, 0.053)	14%

© 2022 Desai P et al. *JAMA Network Open*.

*PA: Low activity (responded to at least four items and reported no activity for all responses), Medium activity (< 150 mins/ wk), High activity (≥150 mins/ wk)
Serum NfL: Low (≤ 25.5 pg/mL), High (> 25.5 pg/mL)
All models adjusted for age, race, sex, education, chronic conditions, and APOE-ε4 allele.

© 2022 Desai P et al. *JAMA Network Open*.
